# Evaluation Time and Efficacy of Root Canal Rotary Preparation in Primary Teeth: An In-Vitro Study

**DOI:** 10.7759/cureus.24558

**Published:** 2022-04-28

**Authors:** Anas H Bnaiyan, Mohamed K Altinawi, Thuraya Lazkani, Hasan Alzoubi

**Affiliations:** 1 Department of Pedodontics and Preventive Dentistry, Damascus University, Damascus, SYR; 2 Department of Restorative Dentistry and Endodontics, Damascus University, Damascus, SYR

**Keywords:** rotary preparation, k-file, baby fanta, primary molars, pulpectomy

## Abstract

Purpose

The objective of this study was to evaluate the efficacy, apical debris extrusion, and time of rotary preparation of Baby Fanta files (Fanta Dental, (Shanghai, China) in root canals of primary molars.

Materials and methods

The study sample consisted of 30 upper secondary molars that were randomly divided into two groups (Baby Fanta - K file, Shanghai, China), each group comprising 15 palatal roots that were prepared, the necessary preparation time was calculated, the apical debris was collected, and they underwent a clearing technique to evaluate the cleaning effectiveness of the files used in this study.

Results

The results showed that Baby Fanta files were superior to K-file in time of preparation (P-value=0.000) while there were no statistical differences in the effectiveness of cleaning (P-value=0.188) or apical debris extrusion (P-value=0.451).

Conclusion

Baby Fanta files can be used effectively for the rotary preparation of root canals in primary teeth.

## Introduction

Pediatric dentistry is one of the most important specialties of dentistry. The goals that pediatric dentistry seeks to achieve are still not sufficiently practiced despite awareness of the modern concepts of pediatric dentistry. Improper treatments for primary teeth lead to problems in chewing, nutrition, and the development of the child at an important stage of his life, which may affect his social behavior [1].

Preserving the primary teeth until their physiological fall is one of the most important goals of pediatric dentistry, to maintain the natural function, aesthetic aspects, correct pronunciation of letters, correct growth, and preservation of the appropriate place for the eruption of permanent teeth later [2].

Treatment of severely infected primary teeth is considered a difficult challenge and has limited treatment options. These options include either extraction of the affected tooth and replacing it with a space maintainer (if required) or performing endodontic treatment (if possible) [3].

The American Academy of Pediatric Dentistry (AAPD) defines a pulpectomy as the process in which we remove the roof of the endodontic chamber to obtain entrance to the root canals. These canals are then prepared, disinfected, and later filled with absorbable material. Thus, the tooth can be preserved within the dental arch without containing any pulp tissue [4].

Pulpectomy in children has high success rates, which made dentists prefer them over tooth extraction [5]. With the recent development in pediatric dentistry and endodontic treatment, nickel-titanium rotary instruments were introduced, which made endodontic treatment easier and faster compared to manual instruments where their use led to the formation of precisely shaped canals. The idea of ​​preparing root canals in primary teeth was put forward by Bar et al. (2000) [6].

Companies have rushed to develop appropriate systems for endodontic treatment in primary teeth and to enhance the accuracy and speed of treatment. Among these systems is the Baby Fanta system from the Chinese company Fanta Dental (Shanghai), and due to the lack of studies on this system, it was necessary to conduct an experimental in-vitro study to evaluate its effectiveness in the endodontic treatment of primary teeth.

## Materials and methods

A randomized controlled experimental in-vitro study was conducted in 30 primary molars to study the timing and efficacy of root canal preparation in primary teeth by using Baby Fanta files (25/04) in comparison with manual preparation by using K-files. The study protocol was approved by the Scientific Research and Postgraduate Board of Damascus University Ethics Committee of Damascus University, Damascus, Syria (IRB No.UDDS-531-18112019/SRC-1450).

The sample size was determined using a sample size calculation program (PS Power and Sample Size Calculation program, Version 3.0.43, https://biostat.app.vumc.org/wiki/Main/PowerSampleSize). The sample size was calculated using outcomes from Rathi et al., comparing the outcomes of two pediatric rotary endodontic files [7]. Sample size calculation produced a required sample size of 30 primary molars to detect a significant difference (90% power, two-sided 5% significance level). The studied sample was randomly distributed using a lottery, where numbers from 1 to 30 were written on paper cards representing the research cases (the numbers shown in Table 1 carry the results of the randomization of the study sample), and then they were randomly divided into two groups: Group A - 15 primary molars were prepared by using Baby Fanta files (25/04) and Group B - 15 primary molars were prepared by using K-files.

**Table 1:** Randomization results of the study sample

Group A															Group B														
1	3	4	7	11	13	16	19	20	22	23	24	25	28	30	2	5	6	8	9	10	12	14	15	17	18	21	26	27	29

The inclusion criteria were absorption of less than a third of the root, no lateral absorption, absence of fissures, caries, fractures in the root, an average curvature of the palatine root of 10-30 degrees, according to the Schneider protocol, a single canal in the palatine root, and no previous endodontic treatments. While the exclusion criteria were molars in which absorption exceeds the apical third, roots affected by caries or fracture, and roots whose anatomical features have been changed as a result of periodontal disease.

**Figure 1:**
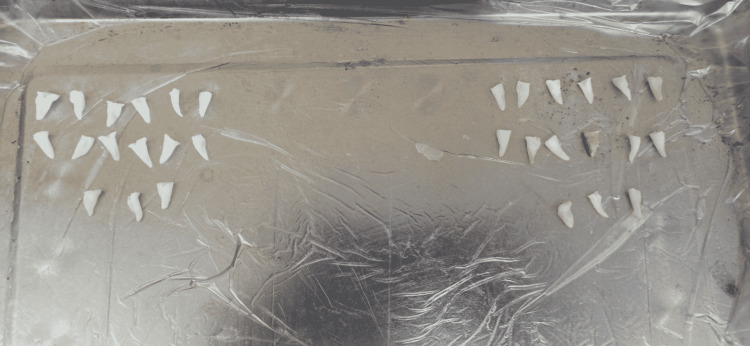
Study sample after standardization of the working length

The sample was immersed in 1% sodium hypochlorite for 15 minutes and then immersed in saline until the start of the study. Coronal access was made by using diamond bur 008 and irrigation by saline. Probing the canals with a 10-15 K-file (Mani, Japan) where canals greater than size 15 were excluded. The working length of all samples was unified by cutting the crown using separation discs to reach a working length of 10 mm (Figure 1).

**Figure 2:**
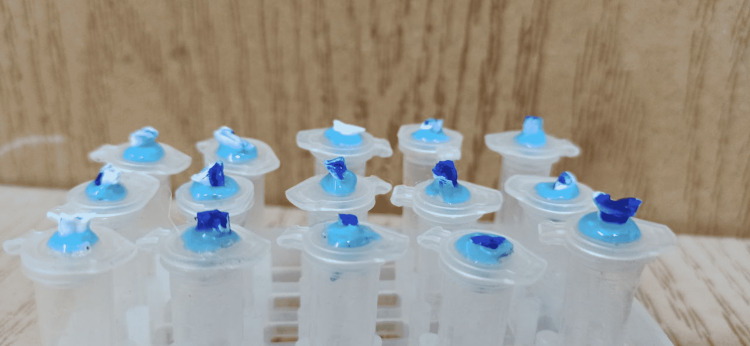
Chinese ink injection

The working length was determined as 1 mm before the apical foramen, depending on the initial file. Two ml of Chinese ink was injected using a 30-gauge insulin syringe three times per canal (Figure 2). The rotary preparation was carried out by using Baby Fanta 25/04 files at a speed of 300 rpm and a torque of 200 g/cm (according to the manufacturer's instructions). The canal was prepared with a 20/04 file and then 25/04 to the full working length and irrigated with saline after each file. The canals were prepared manually by using the crown-down technique so that the apical foramen was prepared up to K-file size 25.

Study of apical extrusion of debris

**Figure 3:**
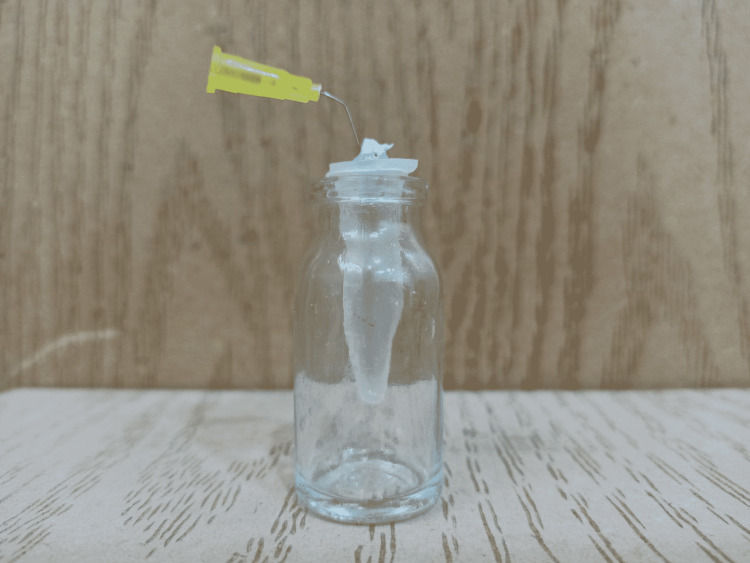
Example work described by the Myers and Montgomery process

Thirty Eppendorf tubes, numbered 1 to 30, were taken and their caps were removed. Three weight measurements were taken for each empty tube and the mean of these readings was recorded as the weight of the empty tube. Then, a hole was punched on each cap, each tooth was inserted into a cap hole, and the space between the tooth and cap hole was sealed with a heatless liquid dam and a block-out resin light cure. A 27-gauge needle was inserted into the side of the cap to equalize the air pressure in and out of the tube. After that, each tube was placed inside a glass vial and the tube was handled only by the vial that holds the tube, and the tubes were not touched with a finger at all (Figure 3). After completing the process of preparing the canals, we separated the cap with the needle and the tooth from the tube, and the adherent residues on the surface of the root were collected by washing the root inside the tube with 1 ml of distilled water. Then, the tubes were placed in an incubator at 70 °C for five days to evaporate the distilled water before weighing the dry residue. After evaporating the distilled water, the tubes containing the dry residues were weighed. The weight of each tube was taken three times and the average value was recorded. The dry weight of the residues was calculated by subtracting the weight of the empty tube from the weight of the same tube containing the residues.

Study of the effectiveness of mechanical cleaning by using the clearing technique

**Figure 4:**
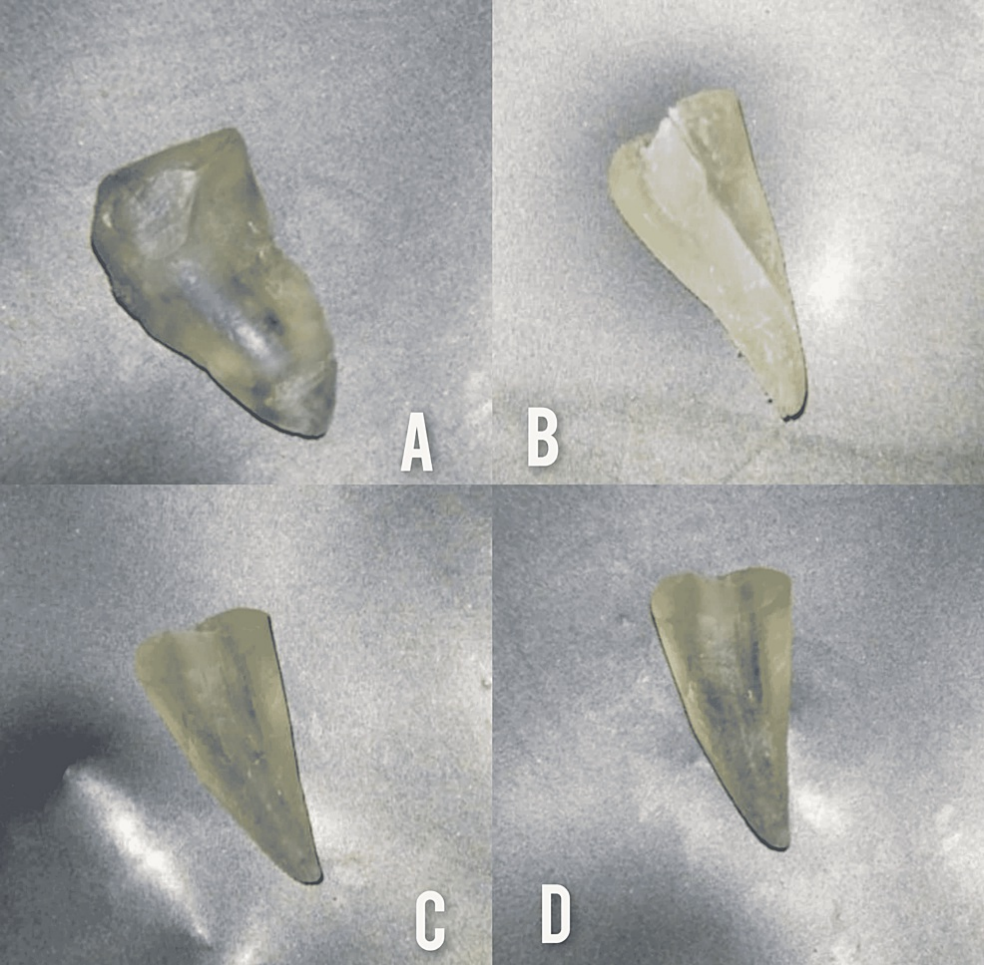
Some of the study samples after the clearing process: (A) Grade 2, (B) Grade 0, (C and D) Grade 1

Teeth were placed in 5% hydrochloric acid for three days, and the acid was replaced every 24 hours, with the solution being stirred four times a day. After that, the teeth were washed with running water for 12 hours to remove traces of acid. Then the teeth were placed in solutions of increasing concentrations of ethyl alcohol in the order of (75%, 85%, 96%, 100%) for four hours each. After that, the molars were placed in methyl salicylate for two hours, the molars were raised, where they appeared completely transparent with the exception of the Chinese ink according to the degree of mechanical effectiveness of the endodontic files used (Figure 4). After performing the clearing process, the Chinese ink in the coronal, middle, and apical region was examined by three people "without prior knowledge of the type of preparation in order to achieve the principle of blinding" by using a light microscope with a magnification of 10x, and the results were recorded according to the following index: Grade 0 - full clearing (the canal is completely clean and no trace of Chinese ink), Grade 1 - almost complete removal of Chinese ink (dots of ink within the canal), Grade 2 - partial removal of Chinese ink (linear areas of ink appear within the canal), and Grade 3 - not removing any part of the Chinese ink.

Measurement of preparation time

The total preparation time, including irrigation time and instrument exchange, was measured using a timer for each group separately.

## Results

The sample consisted of 30 palatal root canals of 30 primary upper second molars that were divided into two equal main groups according to the preparation system used (Baby Fanta Taper 04 rotary preparation system 50%, crown-down manual preparation system 50%).

Studying the effect of the preparation system on the preparation time in the sample

The arithmetic mean preparation time for the Baby Fanta system was 62.20 seconds as compared to 118.73 seconds for the manual system. A T-test was conducted to study the significance of the differences in the average preparation time (in seconds) between the groups in the sample. It is noted that the significance level value is much smaller than 0.05; that is, at the 95% confidence level, there were statistically significant differences in the average values ​​of preparation time (in seconds) between the groups, and since the algebraic sign of the difference between the two averages is negative, we concluded that the values ​​of preparation time in the group Baby Fanta Taper 04 were smaller than in the crown-down manual preparation system in the sample as seen in Table 2.

**Table 2:** Results of preparation time.

The average preparation time for the Baby Fanta system	The average preparation time for the manual system	The difference between the two averages	Calculated t value	p-value
62.20	118.73	-56.53	-21.715	0.000

Studying the effect of the preparation system on the apical extrusion of debris in the sample

The arithmetic mean apical extrusion of debris for the Baby Fanta system was 0.67 mg compared to 0.91 mg for the manual system. A T-test was conducted to study the significance of the differences in the average values ​​of the apical extrusion of debris between the groups in the sample. It is noted that the significance level value is much greater than the value of 0.05; that is, at the 95% confidence level, there were no statistically significant differences as seen in Table 3.

**Table 3:** Results of apical extrusion debris

The average apical extrusion debris for the Baby Fanta system	The average apical extrusion debris for the manual system	The difference between the two averages	Calculated t value	p-value
0.67	0.91	-0.024	-0.764	0.451

Studying the effect of the preparation system on the effectiveness of mechanical cleaning in the sample

A Mann-Whitney U test was conducted to study the significance of the differences in the effectiveness of mechanical cleaning between the groups in the sample. It is noted that the significance level value is much greater than the value of 0.05; that is, at the 95% confidence level, there were no statistically significant differences as seen in Table 4.

**Table 4:** Results of canal cleaning according to the clearing technique

U-value	p-value	The significance of differences
87.0	0.188	No statistically significant

## Discussion

One of the most important goals of endodontic treatment is to eliminate microorganisms from the root canals, and this is achieved by removing pulp tissues, remnants of necrotic pulp, and infected dentin. In the current study, the cleaning efficiency of both rotary and manual preparation methods was analyzed by microscopy [8].

Several studies have compared the cleaning effectiveness of rotary and manual files in primary teeth. There are different techniques to determine the effectiveness of cleaning such as clearing technique, CT scan, quantitative tomography, cone-beam computed tomography (CBCT), scanning electron microscopy (SEM), and stereomicroscopy [9].

Tooth assessment after clearing is a reliable technique for 3D root canal examination, and it is an inexpensive method when compared to other methods according to Tomar et al. [10]. The used Baby Fanta files have a triangular cross-section and are characterized by being heat-treated and having shaped memory, as the heat-treated files are less susceptible to deformation, have the ability to follow the original anatomical shape of the root canals, and the length of the file is 16 mm., This design reduces the contact areas between the file and the dentin, thus reducing stress on the files [11].

Distilled water was chosen as the irrigant in this study to prevent the possible change in weight that could occur due to the crystallization of sodium hypochlorite into sodium crystals [12]. Floral foam was used to simulate the periapical region in a few previous studies [13] but periapical tissues were not simulated in the current study because this method might alter the results, as the apical residuals could be absorbed.

Irrigation was made by using side-hole needles because a regular needle pushes a large volume of irrigant [13]. This was confirmed by Gungor and Kustarci who found that the use of closed-top needles with a side opening causes a lower apical extrusion of the irrigant [14], and this was confirmed by Boutsioukis et al. when comparing several models of irrigation needles and their effect on the apical extrusion of irrigant [15].

In the current study, the palatine root of the primary upper molars was chosen for several reasons [16]: It has a wide root canal, regular shape, and fewer intracanal branches compared to the mesial and distal roots. Where the mesial and distal roots were excluded because they contain anatomical abnormalities that may lead to difficulty in applying the search criteria, as the palatine root was selected in several studies carried out on primary teeth with the aim of evaluating the cleaning effectiveness and the apically extrusion debris [16-17].

The working length of all canals has been standardized because different lengths of canals are associated with a different preparation space for endodontic instruments, and thus a different amount of extruded canal debris. De-Deus et al., in their study on permanent teeth, when comparing several rotary systems, found a difference in the apical extrusion debris according to the lengths of the prepared canals [18].

In the work described by Myers and Montgomery [19], this example was used to collect debris in the primary and permanent teeth and was characterized by simplicity with the possibility of separating the debris from the irrigant to obtain the net weight of the residue.

Within the current study, there were no statistically significant differences in the effectiveness of cleaning between the used files, and this finding is in accordance with the results obtained by Silva et al. [20], Bahrololoomi et al. [21], and Seraj et al. [22]. The results of this study also showed a statistically significant shorter working time for rotary files compared to manual files, and this is in agreement with the study of Barr et al., Ochoa-Romero et al., and Silva et al. [8,20,23]. In contrast to this study, Madan et al. showed that manual files have less canal preparation time than hand files [24].

Factors affecting apical extrusion debris include the size and length of the root canal, the type and size of the endodontic instrument, the point at which the endpoint preparation ended, and the type and amount of irrigant used [12]. The results of this study showed that all canal preparation systems caused apical extrusion debris, which in turn agrees with the results of several studies [25-28].

However, there were no statistically significant differences in the apical extrusion debris between rotary and manual files, and this did not agree with the results of Buldur et al. and Preethy et al. [27,29]. This can be attributed to the difference in the preparation technique used in the manual files, the crown-down technique was used in the current study instead of the step-back technique, where the early coronal expansion secures the removal of the largest number of residues, thus reducing the pushing of these residues out of the apical foramen.

## Conclusions

There were no differences between manual and Baby Fanta files in terms of apical extrusion debris and cleaning efficacy. While a difference was observed in terms of preparation time in favor of Baby Fanta files, Baby Fanta files can be used effectively for the rotary preparation of root canals in primary teeth.
